# Small non‐coding RNA transcriptomic profiling of neural cell‐types in Alzheimer's disease model mice

**DOI:** 10.1002/alz70855_106595

**Published:** 2025-12-24

**Authors:** Nicholas F Fitz, MD Shahnu Alam, Iliya Lefterov, Radosveta Koldamova

**Affiliations:** ^1^ University of Pittsburgh, Pittsburgh, PA, USA

## Abstract

**Background:**

While small non‐coding RNAs (sncRNAs) make up about 1% of the total genome, they maintain a significant role in regulating cellular processes and gene expression. Given the complexity of the brain and the neural cellular responses to Alzheimer's disease (AD) pathologies, sncRNAs likely hold particular importance. This is especially true when considering the coordination of activities between different cell types and disease associated subtypes of glial cells which could potentially be modulated by sncRNAs. We hypothesize that AD model mice will exhibit unique sncRNAs signatures that are neural cell type specific and provide details related to underlying disease mechanisms.

**Method:**

Mouse brains from APP/PS1de9 and wildtype mice were dissociated, and microglia, astrocytes and neurons sorted utilizing magnetic activated cell sorting. Subsequently, both sncRNA and mRNA were isolated and individual libraries generated and sequenced. sncRNAs alignment to the mouse microRNAs (miRNAs), small nucleolar RNAs (snoRNAs), transfer RNA (tRNA), small nuclear RNAs (snRNAs), and circular RNAs (circRNAs) were performed utilizing COMPRSA and differential expression with DESeq2. While mRNA alignment and differential expression was performed with edgeR pipeline.

**Results:**

We present both the sncRNA and mRNA changes in astrocytes, microglia and neurons of AD model mice. We show high level of alignment to the all the subclasses of sncRNAs for all neural cell types, with a high abundance of circRNAs. This expression patterns show unique signatures in the AD model mice compared to their wildtype controls. Integrative modeling of sncRNA binding, targets and associated genes demonstrated that these expression patterns were associated with gene transcriptional responses in the AD model mice. These associations were related to significant changes in gene primarily associated with inflammatory response, metabolic functions and developmental functions.

**Conclusion:**

We observed unique sncRNA signatures in astrocytes, microglia and neurons that were correlated with gene transcriptional changes and association with amyloid pathology. This study is a valuable resource for exploring the role of sncRNA in different neural cell types, unique sncRNA signatures associated with AD like pathologies and their potential use as biomarkers in AD.

